# Lifestyle Intervention Randomized Controlled Trial for Age-Related Macular Degeneration (AMD-Life): Study Design

**DOI:** 10.3390/nu15030602

**Published:** 2023-01-24

**Authors:** Alexandra P. M. de Koning-Backus, Jessica C. Kiefte-de Jong, Jeroen G. J. van Rooij, AMD-Life Team, André G. Uitterlinden, Trudy G. Voortman, Magda A. Meester-Smoor, Caroline C. W. Klaver

**Affiliations:** 1 Department of Ophthalmology, Erasmus MC University Medical Center Rotterdam, P.O. Box 2040, 3000 CA Rotterdam, The Netherlands; 2 Department of Epidemiology, Erasmus MC University Medical Center Rotterdam, P.O. Box 2040, 3000 CA Rotterdam, The Netherlands; 3 Department of Public Health and Primary Care, Health Campus The Hague, Leiden University Medical Center, 2333 ZA The Hague, The Netherlands; 4 Department of Internal Medicine, Erasmus MC University Medical Center Rotterdam, P.O. Box 2040, 3000 CA Rotterdam, The Netherlands; 5 AMD-Life Team, Erasmus MC University Medical Center Rotterdam, P.O. Box 2040, 3000 CA Rotterdam, The Netherlands; 6 Division of Human Nutrition and Health, Wageningen University & Research, P.O. Box 17, 6700 AA Wageningen, The Netherlands; 7 Department of Ophthalmology, Radboud University Medical Center, P.O. Box 9101, 6500 HB Nijmegen, The Netherlands; 8 Institute of Molecular and Clinical Ophthalmology, University of Basel, CH-4031 Basel, Switzerland

**Keywords:** age-related macular degeneration, lifestyle, nutrients, Mediterranean diet, genetic testing, personalized risk-profiling, gut microbiome, behavioral change technique, coaching

## Abstract

Age-related macular degeneration (AMD) has a strong genetic basis, but environmental factors such as smoking and a healthy diet can decrease the genetic fate by up to 50%. Current guidelines for clinical management include recommendations for a healthy lifestyle and antioxidant supplementation. However, many ophthalmologists do not inform their patients of this AMD-beneficial lifestyle. An important reason is the lack of trust that transition of lifestyle will be feasible in persons of advanced age and lack of methodology to measure lifestyle or its biological effects. To address these issues, we set up the lifestyle intervention study AMD-Life. It aims to investigate whether personalized risk-profiling (including genetic testing) and/or additional coaching can motivate patients to change their lifestyle. It also explores which biomarkers best reflect lifestyle change beneficial for AMD. The first year is a three-arm, self-contained open-label randomized clinical trial. A total of 150 AMD patients aged 55–85 years were randomized into three arms: (A) merely standard recommendations; (B) A conditions plus personalized risk profiling based on genetics and lifestyle, (C) B conditions plus coaching. The second year tests sustainability of lifestyle changes without active intervention. AMD-Life can provide further insight into the relevance of these interventions for the clinical management of AMD.

## 1. Introduction

Age-related macular degeneration (AMD) is a leading cause of blindness in the elderly of the Western world. Currently, more than 196 million people worldwide have AMD, and global aging will increase the number of affected persons to 288 million by the year 2040 [1,2]. The prevalence of early and intermediate AMD is ~20% in people aged over 65 years. These stages are characterized by the presence of yellow-white deposits (drusen) under the retinal pigment epithelium (RPE) and/or pigment changes, usually occurring without visual implications. The prevalence of the vision-disabling late AMD ranges from 3% in individuals aged 65 years to ~13% in those over 90 years [3]. This stage is characterized by either macular neovascularization (MNV, neovascular or wet AMD) or loss of RPE and photoreceptors (geographic atrophy, GA, or dry AMD) in the macular area. The transition from intermediate to the late stage of the disease occurs with a frequency of 3–6% per year [4]. Within 10 years, this will increase up to ~60%. The current therapeutic options for wet AMD are anti-VEGF injections, which improve vision drastically in the short term but often fail to protect against severe vision loss in the long term [5]. Therapy for GA has recently made progress with regimens controlling the complement pathway, but this still needs confirmation in real-world studies [6,7].

The genetic architecture of AMD has been mostly uncovered. Several years ago, a large genome-wide association study reported association with 52 common genetic variants in 34 loci [7]. The variants represented prominent pathways such as the complement cascade, lipid homeostasis, and extra-cellular matrix. A genetic risk score (GRS) that aggregated risks of these common variants showed a discriminative accuracy of 0.84 to correctly identify a person with late AMD [8]. In addition to common variants, many rare variants have been identified in genes involved in complements [9], collagen [10], coagulation pathways, and metalloproteinases [11]. Remarkably, although AMD is now one of the best genetically characterized complex diseases [7], genetic testing for AMD is still mostly a scientific enterprise and not a routine assessment in clinical practice. An important reason for this is the position of ophthalmic professional organizations, which do not advise genetic testing for AMD until treatment for specific disease-associated genotypes has been established.

Environmental factors also play a significant role in disease pathogenesis; in particular, smoking [12,13,14,15], dietary patterns [16,17,18], and physical activity [19] have been associated. We recently showed that persons in the most unfavorable tertile of these lifestyle variables had an increased risk of late AMD by at least two-fold [8]. Reduction of the risk is possible with nutrients that have antioxidative properties and polyunsaturated omega-3 fatty acids [16,20,21]. International dietary guidelines (United States, Canada, Germany, France, United Kingdom, The Netherlands, Japan, e.g.) recommend less meat and more plant-based dietary patterns including vegetables, fruit, whole grains, legumes, nuts and seeds, and (fatty) fish [22]. These recommendations are comparable with the Mediterranean diet pattern [23,24] and are also in line with the advice of the UN Food and Agriculture Organization and the World Health Organization [22,25]. However, data show that adherence to these recommendations is low [16,26,27]. In the Rotterdam Study, we recently investigated recommended minimum intake values (RMIV) of vegetables, fruit, and fish and the risk of AMD [16]. We found that only 4% of the participants consumed the recommended amounts of foods. However, those who did showed a significant decreased hazard ratio by more than half of incidence of advanced AMD. Two large clinical trials (AREDS 1; AREDS 2) provided evidence that supplementation with antioxidants and zinc can also significantly reduce disease progression [28,29]. Novel is the finding that intestinal microbiota [30] are associated with AMD, which may mediate the association with dietary factors [5,17,30], oral supplements [29], and other environmental factors [29].

Most preferred practice patterns for AMD recommend lifestyle measures, but the information provided to patients and adherence tend to be poor. Up to 60% of patients do not take oral supplements or use them incorrectly, and smoking cessation or long-term dietary changes are often not achieved [31,32,33,34]. One reason may be that retinal specialists do not convey the message adequately due to limited time, knowledge, or doubts about effectiveness [33,34,35,36,37]. This refutes the current view that healthcare professionals should play a significant role in motivating patients to change habitual patterns or behaviors that pose significant health risks. How does one motivate patients to take action and change behavior? A recent insight is that, at certain times, patients appear to be more receptive to behavioral change and lifestyle advice than usual [36,37], for example, when a disease has been diagnosed, they have received abnormal test results, or have been confronted with a high genetic risk. These are called ‘teachable moments’ and may be considered as optimal timing to start lifestyle counselling [36,37].

In the current AMD-Life trial, we aim to investigate which strategy is most successful in motivating patients to change the behavior that is driving them to vision loss: receiving only general information on risks and benefits, receiving detailed information about personalized risks and targets for change, or receiving coaching based on behavioral change technique (BCT) [38,39] and motivational interviewing (MI) [40,41]. We also aim to explore whether biomarkers in blood and the gut microbiome can be used to monitor lifestyle and lifestyle change, which can then be used to motivate patients further. This article provides the rationale and design of the study.

## 2. Materials and Methods

### 2.1. Study Design and Population

AMD-Life is a 2-year lifestyle intervention trial. The first year is a self-contained open-label randomized clinical trial with 3 intervention arms. The second year aims to investigate the sustainability of improved lifestyle without active intervention. Patients visit the research center 3 times: at baseline, month 12, and month 24. Ethical approval was obtained from the Medical Ethical Committee at Erasmus MC (MEC-2018-063). AMD-Life is registered at ClinicalTrials.gov (registration identifier: NCT05667441).

This study is designed as an inception phase study. We set up the main elements, test our assumptions and feasibility, check procedures, and focus on adherence, reporting participant and physician experience for the three different strategies tested, specifically targeting long-term behavior change.

Sample size calculation indicated that 50 eligible subjects per group would be sufficient to detect an effect size of 0.25 for adherence to lifestyle recommendations after the 12-month intervention (alpha: 0.05, power: (1-beta err prob.): 0.8897, calculated by G*Power).

Recruitment for the study started 2 August 2021 and is expected to end 1 July 2023. Participants are made aware of the study by their attending physician at outpatient clinics (OPC) of Erasmus MC Rotterdam, the Rotterdam Eye Hospital, Radboudumc, in Nijmegen, and other hospitals in the Netherlands. Interested candidates can apply online through the website: www.maculadegeneratie.nl (accessed on January 2023) with the apply button for participation, or by email or telephone.

Inclusion criteria: age between 55 years and 85 years, a diagnosis of intermediate AMD or unilateral late AMD, as determined by multimodal imaging and approved by the EyeNED Reading Center.

Exclusion criteria: participation in other intervention studies for AMD; living in retirement homes (because of difficulty in implementation of diet); dementia; macular pathology other than AMD; liver and kidney insufficiency; persons who are illiterate and have no independent trusted person with them to explain the informed consent form.

Prior to the study, all potential participants are informed and asked to provide written consent. Subsequently, participants are invited to visit the research center. Eligible participants are randomly assigned using a block-wise randomization procedure (in a 1:1:1 ratio) to one of three study arms. The assignment is by means of a computer-generated table with a random number sequence. This table gives a random order of allocation to the ‘Treatment Group’. The order is by date and time of the first study visit and stratified by gender and age categories (55–64, 65–74, 75–85). The first 125 participants are randomized per block (sets) of 6, and the remaining 25 per sets of 3. The researchers are blinded for randomization.

### 2.2. Interventions

**Figure 1:**
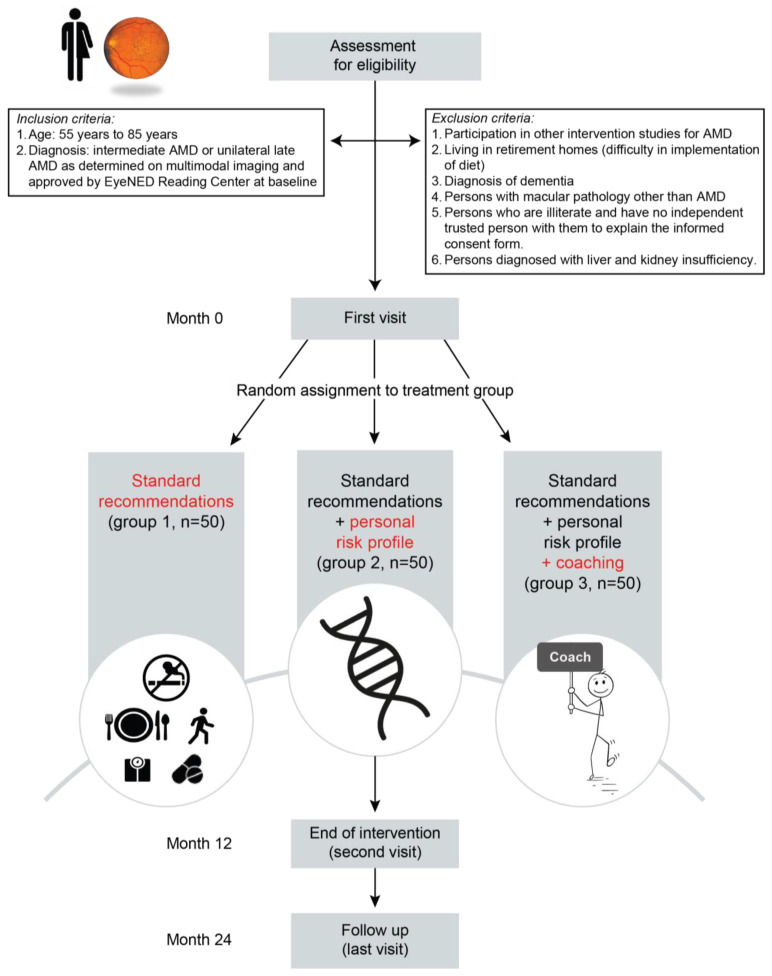
Flow-chart of the AMD-Life study design. Inclusion and follow-up of study participants in each study arm. Three study arms test different strategies to stimulate and increase adherence to a healthy lifestyle. After 12 months, the study arms end, and a single follow-up moment at 24 months is scheduled for assessment of sustainability of lifestyle change.

The AMD-Life study tests three study arms, which each represent a different strategy to stimulate and increase adherence to a healthy lifestyle: (A) patients receive standard recommendations and supplements free of charge; (B) as in A, but patients additionally receive personal risk profiling based on phenotype as well as genetic and environmental risk factors; (C) as in B, but patients additionally receive professional coaching. The schematic overview of inclusion, exclusion, the three arms, and the follow-up is shown in Figure 1.

#### 2.2.1. Group A

Standard recommendations plus AREDS 2 and Omega 3 supplementation (‘standard care’)—n = 50. At the baseline visit, an experienced trial nurse informs the participant of personal outcomes of the measurements performed at the research center and of the standard AMD lifestyle recommendations. Participants also receive this information in a letter. The recommendations include refrain from smoking, perform regular physical exercise, strive for BMI < 25, and increase the intake of the dietary food groups of (green leafy) vegetables, fruit, (fatty) fish, and legumes. The Mediterranean Diet pattern is used as guidance for recommendations (for a more detailed description see Table 1). Participants receive oral supplements with antioxidants according to the AREDS 2 formula and Omega 3 forte fish oil free of charge (Table S1).

#### 2.2.2. Group B

Standard care plus personalized risk profiling—n = 50. Participants receive a personalized risk profile for motivational purposes. They are informed about their AMD genetic risk (low, medium, high, very high) based on the GRS, risk of progression, and the potential gain from lifestyle improvement. The four lifestyle factors of smoking, BMI, diet, and physical exercise are graded using a 13-point easy-to-use lifestyle scoring form (Figure S1 - page 1); participants receive their personal grades and can check their room for improvement.

#### 2.2.3. Group C

**Figure 2:**
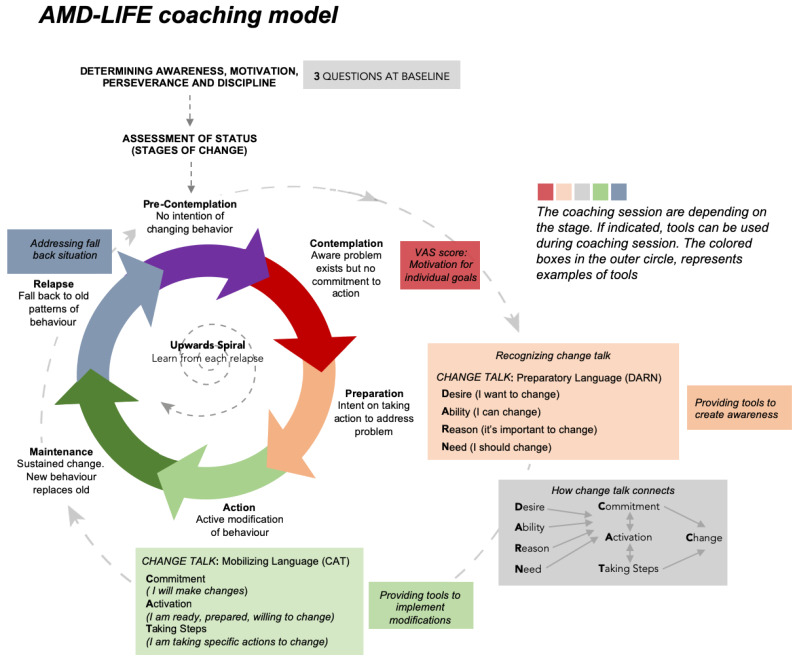
AMD-Life coaching model. The AMD-Life coaching model reflects an upwards spiral to learn from each relapse. Included in the model are: ‘the 3 questions’ at baseline to determine awareness, motivation, perseverance and discipline, and goal-setting; a model for Behavioral Change Technique (BCT), the transtheoretical model of change (determining of ambivalence/assessment of status), adapted with permission from Prochaska and DiClemente [39,41]; and a model for motivational interviewing (MI), increasing/recognizing of ‘change talk’ through DARN-CAT, adapted with permission from Miller and Rollnick [40]. Depending on the stage and/or need of the participant, several tools, information leaflets, documents with assignments, and tables (e.g., specific foods and levels/concentrations of beneficial AMD-nutrients) can be used and/or provided to enhance awareness and stimulate habitual change.

Standard care plus personal risk profiling plus coaching—n = 50. Participants receive coaching based on MI and the stages of BCTs using the AMD-Life coaching model (Figure 2): determination of awareness, motivation, perseverance and discipline, and goal setting. Depending on the stage and/or need of the participant, several tools, information leaflets, documents with assignments, and tables (e.g., specific foods and levels/concentrations of beneficial AMD-nutrients) are provided to the participant to enhance awareness and stimulate habitual change. Coaching sessions take place online by a 15–20 min videoconference twice a month for the first 2 months, followed by once a month for the following 10 months.

After an intervention of 12 months, all study participants visit the research center, after which the participants form a single group to observe the sustainability of lifestyle improvements. At this point, Group A receives insights into their lifestyle changes over the first year and is informed about their genetic risk, similarly to groups B and C, who received this information at month 0 (Figures S1 and S2). At month 24, the study ends with a third visit to the research center.

### 2.3. Avoiding Attrition Bias

Multiple strategies are employed to reduce attrition in all three arms, including an effective recruitment process, brief and informative encounters with staff members, a good tracking system to facilitate participant contact, anti-oxidant and fish oil supplementation free of charge, and the development of a trusting and collaborative relationship between researchers and participants.

If a participant wishes to withdraw from the study intervention, a telephone call is made prior to withdrawal to discuss the reason and possible solutions. If there is no solution, the reason for withdrawal is documented in the participant records for the subsequent analysis and the interpretation of the results.

### 2.4. Data Collection and Measurement

**Figure 3:**
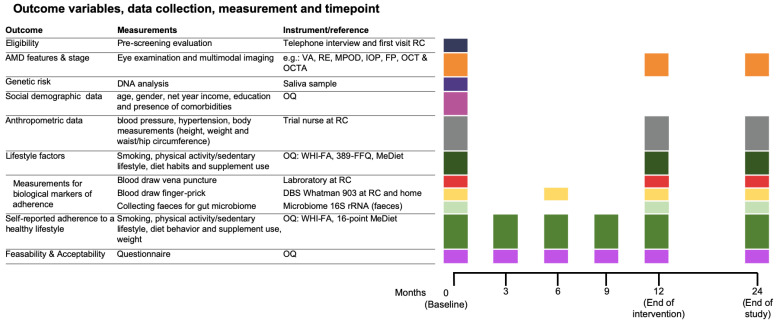
Data collection in AMD-Life. All study participants have the same measurements, irrespective of the study arm. Abbreviations: RC, research center; AMD, age-related macular degeneration; VA, best-corrected visual acuity; RE, refractive error; MPOD, Macular Pigment Optical Density; IOP, intra-ocular pressure; FP, fundus photography; OCT, optical coherence tomography; OQ, online questionnaire; WHI-FA, Women’s Health Initiative Physical Activity Questionnaire; 389-FFQ, food frequency questionnaire; MeDiet, Mediterranean Diet Score; BMI, body mass index; DBS, dried blood spot.

Data collection is performed at month (mo.) 0, 3, 6, 9, 12, and 24. Outcome variables, data collection, measurements, and time points are depicted in Figure 3.

#### 2.4.1. Ophthalmologic Examination

At mos. 0, 12, and 24, participants undergo a comprehensive ophthalmologic examination consisting of measurement of best-corrected visual acuity (VA); refractive error (TopCon TRK-2P objective refractometer); Macular Pigment Optical Density (MPOD; MPS II); intra-ocular pressure (IOP; non-contact tonometer); fundus photography, OCT, and OCTA (3D macula, 3D optic disk, wide, and angiography with T Topcon Triton plus, Topcon 3D OCT 2000-plus; Topcon TRC 50EX with a NIKON D7100 camera).

Features and stages of AMD are classified by grading of multimodal images according to a modification of WARMGS (CFP) [42] and IN-OCT (OCT) consensus by 4 experienced human graders. Quantification of lesions are determined using Deep Learning algorithms developed and available at EyeNED Reading Center [42,43,44,45].

#### 2.4.2. Genetic Factors

Subjects provide a saliva sample for DNA analysis 1 month prior to visiting the research center. Genotyping is performed for known common AMD risk variants, rare variants associated with AMD, and mutations in AMD-mimicking genes. The genotyping platform used is the Illumina Global Screening version 3 MD array with a custom content containing >3500 probes for detecting rare variants and mutations (GSAv3MD-GOALLv1) in CFH (n = 198), CFI (n = 90), C3 (n = 71), C9 (n = 20), ABCA4 (n = 1239), TIMP3 (n = 9), PRPH2 (n = 71), BEST1 (n = 27), CTNNA1 (n = 37), and MFRP (n = 2). Total genetic risk score (GRS) for common variants is calculated by the following formula for GRS, where Gi represents the genotype of variant i and βi represents the effect size of variant i (natural logarithm of the fully conditioned odds ratio of the minor allele of variant i), based on the GWAS of the International Age-Related Macular Degeneration Genomics Consortium [7]. Genotypes were coded as 0, 1, or 2 or, if the genotype is available from imputation, based on the number of minor alleles. Detailed information on SNPs used in GRS calculation is available at (Table S2). GRS is subsequently stratified into tertiles based on the distribution of GRS in the EYE-RISK cohort as Low (GRS < −0.057), Intermediate (GRS > −0.057 & < 1.131), or High (GRS > 1.1131) genetic risk [8]. High GRS is then stratified in equal proportion into High and Very High (GRS ≥ 3).

#### 2.4.3. Covariates

All questionnaires used in the AMD-Life study are digital/online. The participants receive an e-mail with a secure link to the online questionnaires, and data are entered directly into the AMD-Life database.

##### Social Demographics

We ask about baseline social demographic factors by means of a short questionnaire at mo. 0. This includes gender, age, education, eye disorders, family history of AMD, occupation, presence of comorbidity (such as diabetes, cardiovascular disease, hypertension), and living area (6-digit postal code).

##### Anthropometric Data

Anthropometric data are measured and collected at the research center at mos. 0, 12, and 24. Body measurements, including height, weight, and waist and hip circumference, are measured in kilograms and centimeters. Weight and height are used to estimate the body mass index (BMI = weight/(height)2). Blood pressure, systolic and diastolic, are measured, using an automatic sphygmomanometer. Hypertension is defined as a systolic blood pressure ≥ 140 mmHg or a diastolic blood pressure ≥ 90 mmHg. 

#### 2.4.4. Biochemical Measurements

To explore whether biological markers in body specimen are associated with lifestyle change and may be used as a guide star, we draw blood by vena puncture (mos. 0, 12, 24) and by finger-prick (mos. 0, 6, 12, 24), and ask the participant to collect feces (mos. 0, 12, 24).

##### Vena Puncture

Biochemical measurements such as anti-oxidants, fat soluble vitamins, minerals, fatty acids, lipids, complement factors, and inflammatory markers are sampled from serum, EDTA plasma, and whole blood obtained by vena puncture. All samples are prepared for each required analysis according to standard methods. Aliquots are stored (−80 °C) for later analysis.

##### Finger-Prick

To assess whether results from finger prick measurements performed at home may also be used as biological markers of adherence, we ask participants to collect dried whole blood spots (DBS) on a 903 protein saver card from Whatman (mo. 6 at home, mos. 0, 12, 24 at RC) [46,47]. Finger-prick biomarkers assays include anti-oxidants, fat soluble vitamins, minerals, and fatty acids.

##### Collecting Feces

To measure diversity and composition (i.e., alpha and beta diversity and single taxa) of the fecal microbiome over time, we ask participants to collect a sample of their feces at home at three time points: mos. 0, 12, and 24. Participants receive a feces collection kit with all necessary materials. The sample is collected using a preservative and stored at –80 °C upon arrival at the research center for later analysis. Medication use (such as antibiotics) is registered as well (yes/no; if yes, which).

#### 2.4.5. Lifestyle Factors

##### Smoking

Smoking habits are assessed by questionnaire at mos. 0, 3, 6, 9, 12, and 24. Questions include smoking of cigarettes, cigars, tobacco pipe: yes, currently/yes, but quit/no never/; year of smoking cessation; number of cigarettes/cigars/pipe per day; number of years of smoking.

##### Dietary Assessment and Supplement Use

A 389-item food frequency questionnaire (389-FFQ) developed for Dutch adults [48,49] is used to measure dietary behavior at mos. 0, 12, and 24. The 389-FFQ records frequency of each intake as times per day, per week, or per month. The portion sizes are in grams per day (g/day). Nutrient intake data are calculated using the Dutch Food Composition Tables [50], in close collaboration with the Department of Human Nutrition, Wageningen University, the Netherlands. The 389-FFQ was validated against other dietary assessment methods, and adequate ranking of participants according to food and nutrient intake was demonstrated [48,49,51,52]. Oral supplement use (yes or no) is measured using self-reported questionnaires at different time points.

Adherence to Mediterranean diet is scored in two ways: (1) analysis of data from the 389-FFQ; (2) analysis of data from the validated 14-point MeDiet screening instrument [53,54] at mos. 0, 3, 6, 9, 12, and 24. The 14-point MeDiet screening instrument is a modified version of the original standardized Mediterranean Diet Score (MDS) [23,24]. We added two additional questions to this instrument in our version (Table 1).

The 16-point MeDiet is used as part of the lifestyle form that is available for groups B and C. In the lifestyle form, outcomes of 13 out of the 16 questions, as indicated in Table 1, are used.

##### Physical Activity

Physical exercise is assessed using the validated ‘Women’s Health Initiative (WHI) Physical Activity Questionnaire’ [55] at mos. 0, 12, and 24. The WHI Physical Activity Questionnaire is a common instrument and has previously been used in several AMD studies [19]. The questionnaire contains questions on walking, cycling, gardening, various sports, and housekeeping, according to time spent in light, moderate, and vigorous activity.

##### Feasibility and Acceptability

To assess feasibility and acceptability, we ask participants about their own experience with AMD, their experience with the recommendations, and depending on the intervention group, we ask about their experience with the personalized risk score and additional coaching (mos. 3, 6, 9, 12, and 24) by a short questionnaire.

##### Assessment of Change in Habitual Behavior

All coaching sessions (Group C) are recorded by video and registered in the AMD-Life database. Reporting includes motivation, goal-setting for change, self-confidence according to the AMD-Life coaching model (which aims to reflect an upwards spiral), and learning from potential relapses (Figure 2). In order to start the coaching process, we ask the participants at baseline to fill out a short questionnaire regarding (1) receiving information; (2) motivation, perseverance, and discipline; and (3) acceptance that unbeneficial but often enjoyable habit patterns need to change. Participants rank their motivation, perseverance, and discipline on a visual analogue scale (VAS) from 0 to 10 (with 0 representing no/lowest, 5 moderate, and 10 highest motivation, perseverance, and/or discipline). Subsequently, individual lifestyle goal(s) on one (or more) of the four lifestyle components (smoking, food pattern, weight, and/or physical activity) are defined. The following steps determine ambivalence/assessment status: the stages of change model (the transtheoretical Model of Change) [39,41]; and the ‘DARN-CAT’ motivational interviewing model (increasing/recognizing of ‘change talk’ using the six signs in the individual’s language) [40,41]. The contents of the coaching depend on the stage and/or need of the participant and lifestyle assessments. Documents with assignments and tables (e.g., specific foods and levels/concentrations of beneficial AMD-nutrients) are provided to enhance awareness and stimulate habitual change during these individual coaching sessions.

To measure, score, and visualize progression or relapse, and to enhance adherence during the coaching sessions, we developed a 13-point easy-to-use lifestyle scoring form (Figure S1) and a visualization form (Figure S2) for follow up. Both forms include the total GRS (Figure S1 page 2) and lifestyle information. Lifestyle risk estimates are based on validated studies (i.e., smoking, BMI, MeDiet (13 items), physical activity). Each healthy outcome receives a point. The total score can range from 0 to 13, in which high scores represent an AMD-healthy lifestyle (Figures S1 and S2).

The lifestyle score form is used at baseline for group B and C, and at each additional time point (mos. 0, 3, 6, 9, 12, and 24) for Group C. At the second (mo. 12) and third (mo. 24) visit at our research center, all participants receive the complete lifestyle score form and visualization thereof at mos. 0, 12, and 24, respectively. For a more detailed description, see the Supplementary Materials.

## 3. Study Outcomes

The study outcomes are as follows: adherence to lifestyle recommendations; identify change over time in biomarkers from blood related to AMD lifestyle determinants; the feasibility and acceptability of participants compared across the three intervention strategies, as based on quantitative and qualitative data.

### 3.1. Primary Study Outcome

Improvement of the overall lifestyle score was measured by the 13-point form (see previous paragraph and Figure S1: Lifestyle scoring form), which is based on the questionnaires inquiring about smoking habits, BMI, MeDiet, and physical activity taken at baseline and 12 months (see previous paragraph data collection and measurement).

### 3.2. Secondary Study Endpoints of AMD-Life

Improvement of the overall lifestyle score at 24 months.

Change of levels of blood biomarkers from serum/plasma and finger-prick at 12 and 24 months.

Personal feasibility and acceptability of lifestyle change measured by questionnaires at 3, 6, 9, 12, and 24 months.

Changes in macular pigment optical density as measured with MPOD at 12 and 24 months.

Changes in quantification of features (e.g., drusen) and stage of AMD as measured on multimodal images at 12 and 24 months.

#### Other Study Parameters

Other study parameters are as follows: age, gender, blood pressure, hypertension, education and socioeconomic factors, comorbidity such as cardiovascular disease, medication, genetic susceptibility of AMD evaluated by GRS, major risk and protective variants, and rare variants.

### 3.3. Statistical Analyses

To compare baseline characteristics between treatment groups we will use the Chi square test and ANOVA. We will conduct longitudinal analysis on the primary and secondary outcomes, with treatment groups as independent variables. To account for clustering within the individual due to repeated measurements, we will use mixed-effects models. We will conduct ‘intention to treat’ analysis and per protocol as a sensitivity analysis.

All statistical analyses will be carried out using R, version 3.5.3 (R Core Team, 2016) and SPSS Statistics, version 29.0.0.0 (IBM, Armonk, NY, USA).

## 4. Discussion

Achieving sustainable lifestyle change in AMD patients is challenging in daily practice. The goal of the AMD-Life study is to define the best strategy to accomplish awareness and sustainable compliance to recommendations by participants diagnosed with AMD. The study is a 1 year, three-arm (group A, B, and C), self-contained open-label randomized clinical trial, followed by an observation period in year 2.

An important strength of our study is the very detailed exploratory investigation, such as the block-wise randomized design, the close monitoring of participants, and the extensive questionnaires about the major lifestyle exposures for AMD, smoking, dietary pattern, BMI, and physical activity. We use the validated 389-dietary FFQ to measure dietary behavior and to record frequency of each intake as times per day, per week, or per month. Thereby, we are able to calculate different dietary pattern scoring methods, such as the Mediterranean diet score to achieve insight on adherence to food categories, and we are able to calculate single nutrient intake. The 16-point MeDiet screening instrument allows quick analysis of food intake and prompt determination of recommendations for change. Smoking is a well-established risk factor, but an infrequent exposure in this age-category; BMI and physical activity are less well established for AMD, but obesity and sedentary behavior are much more prevalent. An approach that combines these four exposures is likely to be more successful than a focus on only one exposure. Other strengths include extensive phenotyping of AMD features and a large set of other covariates for analysis, including biomarkers. The biomarker panel that will be assessed is very extensive, with the goal of exploring associations related to lifestyle and AMD. It involves complement factors in the active and nonactive state, oxidative stress markers, and markers of inflammation, lipids, anti-oxidants, fat soluble vitamins, minerals, and fatty acids. When confirmed, these biomarkers can serve as guidance and motivation for lifestyle change. The patient will be ‘in control’ of his/her own disease and have a better understanding of the consequences for the risks of his/her behavior.

Potential limitations of this study are the relatively small number of study participants. This study is designed as a pilot (inception phase) study. During this phase, we will set up the main elements, test our assumptions and the feasibility, check procedures, focus on adherence, and report participant and physician experience for three different strategies for stimulating a healthy lifestyle in AMD patients, specifically targeted at long-term behavioral change. Another limitation is the use of self-reported physical activity. More objective measurements could be obtained using triaxial accelerometers. A disadvantage of block randomization is that the allocation of participants could result in selection bias [56]. To reduce this bias, investigators are blinded to the randomization procedure.

Meta-analyses show that motivational interviewing (MI) is effective for decreasing alcohol and drug use in adults and adolescents, and evidence is accumulating in other areas of health, including smoking cessation, improving adherence to treatment and medication, and diabetes management [57,58,59,60]. The AMD-Life study will assess if coaching based on the behavioral change technique (BCT) [38,39] and MI [40,41] can be of additional value to change habitual behaviors and adhere to the AMD recommendations. Because implementation of a lifestyle coaching program in AMD management will be challenging, we evaluate changes in habitual behavior and assess adherence at different time points (mos. 3, 6, 9, and 12); this will provide useful information for the implementation of lifestyle coaching programs.

Over 200 clinical trials are taking place targeting various processes in the pathogenic pathway (e.g., complement activation). Aside from the direct costs, these trials also take a toll on patients, which need to be enrolled in large numbers and adhere to these trials without a warranty of benefit. It is not a secret that 90% of drug candidates in clinical trials fail, and patients who have entered trials before are usually excluded from new ones. If trials manage to be successful, new drugs rarely have the potential to reduce the risk of disease progression by half. Lifestyle intervention has this potential. AMD-Life, when validated, will provide the evidence that lifestyle changes can be achieved in persons at risk of AMD progression.

The psychological and social impact of AMD on quality of life is significant, and health-care costs related to vision loss are expected to increase exponentially over the following years [61]. Annual costs of care and treatment of AMD in Europe currently exceed €75 billion; macroeconomic costs related to visual disability can be up to €100 billion. The AMD-Life study fits well within the changing paradigm of patient and healthcare management. In general, clinical focus is moving from medical treatment of end-stage diseases to preventative actions at early stages. Insight into which strategy (A, B, or C) will stimulate preventative actions and adherence to clinical recommendations will not only slow down disease progression but also support wider clinical application of lifestyle intervention and subsequently lower patient burden and health care costs.

## 5. Conclusions

Patients at risk for AMD show low general adherence to healthy diets or regular intake of supplements. ADM-Life was set up to research and assess in clinical application whether adherence improves if patients experience a direct reward or receive feedback of the benefits of their behavior change to general health. The ultimate goal is to reduce the risk of late AMD and thereby improve individual lives and reduce blindness and huge health care costs.

## Figures and Tables

**Table 1:** Mediterranean diet score instrument (MeDiet).

		Criteria for		Included in
Questions		0.5 point	1 Point	LF Scoring
1	Do you use olive oil as the main culinary fat?		Yes	√
2	How much olive oil do you consume per day (incl. oil used for frying, salads, out-of-house meals, etc.)?	2–3 tbsp	≥4 tbsp	√
3	How many grams of vegetable (incl. raw or as a salad) do you consume per day?	200–400 g	≥400 g	√
4	How many fruit units (incl. natural fruit juices) do you consume per day? (units of 80 g each)	2	≥3	√
5	How often do you eat meat?		<1 p/d	
6	If you consume meat, what kind of meat do you consume most of the time? Do you preferentially consume lean meat: chicken, turkey, or rabbit meat, instead of red meat: veal, pork, hamburger, or sausage?	Both equally often	lean meat	√
7	How many servings of meat do you consume per week?		<100–150 g	√
8	How many servings of butter, margarine, or cream do you consume per day? (1 serving: 12 g)		<12 g	√
9	How many sweetened and/or carbonated beverages do you drink per day?		<1 gl	√
10	How many glasses of red wine do you consume per week? (1 glass = 124 mL)		≤3 gl	
11	How many grams of legumes do you consume per week? (450 g per week = 65 g per day)	300–450 g	≥450 g	√
12	How many servings of fish or shellfish do you consume per week? (1 fish = 100–150 g or 4–5 units of shellfish = 200 g)	150–300 g	≥300 g	√
13	How often per week do you consume fatty fish? (Salmon, tuna, herring, eel, sardines, mackerel)		1 p/w	
14	How many times a week do you eat sweets or pastries such as cakes, cookies, and biscuits?		<3	√
15	How many servings of nuts (including peanuts) do you consume per week? (1 serving = 30 g = about a handful)	30–89 g	≥90 g	√
16	How many times per week do you consume vegetables, pasta, rice, or other dishes seasoned with sofrito (sauce made with tomato and onion, leek, or garlic and simmered with olive oil)?	1	≥2	√

Short questionnaire to assess adherence to the 16-point MeDiet. Abbreviations: LF, Lifestyle Form; incl., including; etc., et cetera; tbsp, tablespoon; g, gram; p/w, per week; p/d, per day; gl, glass.

## Data Availability

Data are held in at the Erasmus Medical Center and are available upon written request.

## Supplementary Materials

The following supporting information can be downloaded at: https://www.mdpi.com/article/10.3390/nu15030602/s1, † Summary of the AMD-Life team members; Tabel S1: Oral supplements (AREDS 2 formula & Omega 3 forte fish oil) provided to participants in AMD-Life study for 1 year; Tabel S2: Single Nucleotide Polymorphisms used for AMD-Life GRS calculation; Figure S1: Three page, easy-to-use scoring form used in the AMD-Life study to inform participants on their lifestyle and genetic risk and to stimulate lifestyle change; Figure S2: Visualization of the lifestyle scoring over time. The overview graph visualizes the current status of the lifestyle and enables to display follow up measurement.
