# Supplementary material for: Lifestyle Intervention Randomized Controlled Trial for Age-Related Macular Degeneration (AMD-Life): Study Design
Source: Nutrients. 2023 Jan 24;15(3):602. doi: 10.3390/nu15030602 (PMC9920008; doi:10.3390/nu15030602)
Supplement: Supplementary file 1 [file nutrients-15-00602-s001.zip › nutrients-2150116-supplementary.pdf]

Supplementary material

Alexandra P.M. de Koning-Backus [1,2], Jessica C. Kiefte-de Jong [2,3], Jeroen G.J. van Rooij [2,4], AMD-Life Team [5,†], André G. Uitterlinden [2,4], Trudy G. Voortman [2,6], Magda A. Meester-Smoor [1,2,‡] and Caroline C. W. Klaver [1,2,7,8,‡,*]

[†]AMD-LIFE Team

Corina Brussee [1,2], Amal Hamimida [1,2], Irene van Zeijl [1,2], Eric F. Thee [1,2], Daniël T. Luttikhuizen [1,2], Bart Liefers [1,2], Joëlle E. Vergroesen [1,2], Jeroen P. Vermeulen [1,2,], Annemiek Krijnen [1,2], Marianne van Veen-van der Lelij [1,2], Jeanette Vergeer-Dorp [2], Andrea Vermeij-Verdoold [2], Eugenia Vollebregt-Karamatali [2], Andy van Oosterhout [2], Jose P. Martinez [9], Lambertus P.W.J. van den Heuvel [10], Johannes R. Vingerling [1], Robert Kraaij [4], Bahar Sedaghati-Khayat [4], Tos J.M. Berendschot [11], Monica Barroso [2,12], Janneke J.C. van Lith-Verhoeven [13], Theodorus L. Ponsioen [14], Odette A.M. Tigchelaar-Besling [15], Wishal D. Ramdas [1,2], Wieke Zuidervaart [9]

[1] Department of Ophthalmology, Erasmus MC University Medical Center Rotterdam, PO Box 2040, 3000 CA Rotterdam, The Netherlands
[2] Department of Epidemiology, Erasmus MC University Medical Center Rotterdam, PO Box 2040, 3000 CA Rotterdam, The Netherlands
[3] Department of Public Health and Primary Care, Health Campus The Hague, Leiden University Medical Center, The Hague, The Netherlands
[4] Department of Internal Medicine, Erasmus MC University Medical Center Rotterdam, PO Box 2040, 3000 CA Rotterdam, The Netherlands
[5] AMD-Life team, Erasmus MC University Medical Center Rotterdam, PO Box 2040, 3000 CA Rotterdam, The Netherlands
[6] Division of Human Nutrition and Health, Wageningen University & Research, P.O. Box 17, 6700 AA Wageningen, The Netherlands
[7] Department of Ophthalmology, Radboud University Medical Center, P.O. Box 9101, 6500 HB Nijmegen, The Netherlands
[8] Institute of Molecular and Clinical Ophthalmology, University of Basel, CH-4031 Basel, Switzerland
[9] The Rotterdam Eye Hospital, Rotterdam, The Netherlands
[10] Laboratory for Genetic Endocrine and Metabolic Diseases, Institute for Metabolic and Genetic Disease, Radboud University Medical Centre, 6525 GA Nijmegen, The Netherlands
[11] Department of Ophthalmology, University Eye Clinic Maastricht, 6229 HX Maastricht, The Netherlands
[12] University College Utrecht, Utrecht University, 3584 ED Utrecht, The Netherlands
[13] Department of Ophthalmology, St. Elisabeth Hospital (ETZ), 5022 GC Tilburg, The Netherlands
[14] Department of Ophthalmology, Isala Hospital, 8025 AB Zwolle, The Netherlands
[15] Department of Ophthalmology, Amphia Hospital, 4818 CK Breda, The Netherlands
* Correspondence: c.c.w.klaver@erasmusmc.nl
† The AMD-Life team, members are summarized in the supplementary material.
‡ These authors contributed equally to this work.

Tabel S1. Oral supplements provided to participants in AMD-Life study for 1 year

**AREDS 2 formula**	Composition per daily dose of 2 (soft-gel) capsules:
Vitamin C (L-ascorbic acid)	500mg (625% DRI*)
Vitamin E (DL-alfa-tocoferyl acetate)	400 IU (2233% DRI*)
Zinc (zinc sulfate)	25mg (250% DRI*)
Copper (copper gluconate)	2mg (200% DRI*)
Lutein (from Tagetes erecta)	10mg**
Zeaxanthin (from Tagetes erecta)	2mg**
**Omega 3 forte fish oil 1000 mg**	Composition per daily dose of 2 (soft-gel) capsules:
Eicosapentaenoic acid (EPA)	33%
Docosahexaenoic acid (DHA).	25%
Vitamin E (DL-alfa-tocoferyl acetate)	5 mg (42% DRI*)

** DRI = daily reference intake, ** No DRI established*

Participants will receive their first six months' supply of oral supplements at their first visit at the research center. The second six months' supply will be sent to their home address.

Tabel S2. Single Nucleotide Polymorphisms used for AMD-Life GRS calculation

**chr**	**pos_hg37**	**rsID**	**Gene**	**Reference allele**	**Alternative Allele**	**Beta****	**MAF gnomAD**	**Dosage Source in AMD-Life**
1	196380158	rs187328863	CFH	C	T	0,385262	0,04	Imputed
1	196613173	rs148553336	CFH	T	C	-1,17118	0,01	Genotyped
1	196657064	rs570618	CFH	G	T	0,553885	0,37	Genotyped
1	196704632	rs10922109	CFH	C	A	-0,67334	0,41	Genotyped
1	196706642	rs35292876	CFH	C	T	0,431782	0,01	Imputed
1	196716375	rs121913059	CFH	C	T	3,863463	0,0002	Genotyped
1	196815450	rs61818925	CFH	G	T	0,165514	0,51	Imputed
1	196958651	rs191281603	CFH	C	G	-0,8916	0,0041	Genotyped
2	228086920	rs11884770	COL4A3	C	T	-0,08338	0,28	Genotyped
3	64715155	rs62247658	ADAMTS9	T	C	0,131028	0,42	Imputed
3	99180668	rs140647181	COL8A1	T	C	0,615186	0,03	Imputed
3	99419853	rs55975637	COL8A1	G	A	0,14842	0,11	Genotyped
4	110659067	rs10033900	CFI	C	T	0,139762	0,48	Genotyped
4	110685820	rs141853578	CFI	C	T	1,633154	0,0004	Genotyped
5	35494448	rs114092250	PRLR/SPEF2	G	A	-0,34249	0,03	Imputed
5	39327888	rs62358361	C9	G	T	0,512824	0,01	Genotyped
6	31930462	rs429608 or rs116503776	C2/CFB	G	A	-0,67334	0,14	Genotyped
6	31946792	rs144629244	C2/CFB/SKIV2L	G	A	1,026042	0,01	Imputed
6	31947027	rs181705462	C2/CFB/SKIV2L	G	T	0,444686	0,01	Genotyped
6	32155581	rs114254831	C2	A	G	0,122218	0,26	Imputed

6	43826627	rs943080	C2	T	C	-0,13926	0,48	Genotyped
7	99991548	rs7803454	PILRB	C	T	0,139762	0,19	Genotyped
7	104756326	rs1142	KMT2E/SRPK2	C	T	0,131028	0,36	Genotyped
8	23082971	rs79037040	TNFRSF10A	T	G	-0,11653	0,49	Imputed
9	73438606	rs71507014	TRPM3	C	-	0,10436	0,45	Imputed
9	76617720	rs10781182	MIR6130	G	T	0,113329	0,27	Genotyped
9	101923372	rs1626340	TGFBR1	G	A	-0,12783	0,22	Genotyped
9	107661742	rs2740488	ABCA1	A	C	-0,11653	0,24	Imputed
10	24999593	rs12357257	ARHGAP21	G	A	0,113329	0,26	Genotyped
10	124215565	rs3750846	ARMS2	T	C	1,075002	0,25	Imputed
12	56115778	rs3138141	RDH5/CD63	C	A	0,165514	0,19	Genotyped
12	112132610	rs61941274	ACAD10	G	A	0,470004	0,02	Imputed
13	31821240	rs9564692	B3GALTL	C	T	-0,10536	0,32	Genotyped
14	68769199	rs61985136	RAD51B	T	C	-0,12783	0,47	Genotyped
14	68986999	rs2842339	RAD51B	A	G	0,165514	0,11	Genotyped
15	58680954	rs2043085	LIPC	T	C	0,139762	0,63	Genotyped
15	58723939	rs2070895	LIPC	G	A	-0,15082	0,21	Genotyped
16	56994528	rs17231506	CETP	C	T	0,10436	0,29	Genotyped
16	56997349	rs5817082	CETP	-	A	-0,13926	0,33	Genotyped
16	75234872	rs72802342	CTRB2/CTRB1	C	A	-0,23572	0,08	Genotyped
17	26649724	rs11080055	TMEM97/VTN	C	A	-0,08338	0,51	Genotyped
17	79526821	rs6565597	NPLOC4/TSPAN10	C	T	0,113329	0,37	Imputed
19	1031438	rs67538026	CNN2	C	T	-0,10536	0,43	Imputed
19	5835677	rs12019136	C3 (NRTN/FUT6)	G	A	-0,30111	0,04	Imputed
19	6718146	rs147859257	C3	T	G	1,169381	0,0027	Genotyped
19	6718387	rs2230199	C3	G	C	0,385262	0,15	Genotyped
19	45411941	rs429358	APOE	T	C	-0,40048	0,14	Imputed
19	45748362	rs73036519	APOE	G	C	-0,09431	0,29	Imputed
20	56653725	rs201459901	MMP9	-	A	-0,27444	0,12	Imputed
20	44614992	rs142450006	C20orf85	TTTC	-	-0,17435	0,17	n.a*
22	33105817	rs5754227	SYN3/TIMP3	T	C	-0,23572	0,13	Genotyped
22	38476276	rs8135665	SLC16A8	C	T	0,131028	0,22	Genotyped

* rs152450006 is currently missing in our dataset.
** from Fritsche et al, 2016 [7]

AMD-LIFE Lifestyle scoring form

AMD-LIFE participant number [ ] Study arm (A, B or C) [ ]

SMOKING

Yes	No, recently stopped up to 10 years ago	No, stopped 10-20 years ago	No, never smoked or stopped more than 20 years ago
0	2	3	4

BMI

> 25	≤ 25
0	1

MEDITERRANEAN DIET SCORE

Item			
Olive oil yes = 1	0		1
Olive oil ≥ 4 tbsp / day = 1	0	0.5	1
Vegetables ≥ 400 gr / day = 1	0	0.5	1
Fruit ≥ 3 / day = 1	0	0.5	1
Mainly lean meat = 1	0	0.5	1
Red meat < 1 portion / day = 1	0		1
Butter < 1 portion / day = 1	0		1
Sugery drinks < 1 / day = 1	0		1
Legumes ≥ 450gr / week = 1	0	0.5	1
Fish ≥ 300 gr / week = 1	0	0.5	1
Biscuits/cakes < 3 / week = 1	0		1
Nuts ≥ 90 gr / week = 1	0	0.5	1
Tomato-based food ≥ 2 / week = 1	0	0.5	1

Total score

0-4	5-7	8 - 9	10 -11	12-13
0	1	2	4	5

PHYSICAL ACTIVITY

	Intense	Moderate	Walking
minutes per week			

Total per week

.... minutes = ... hours

< 3 hours/week	3 – 6 hours per week	> 6 - 10 hours per week	> 10 hours per week
0	1	2	3

TOTAL LIFESTYLE SCORE AMD-LIFE (score between 0 en 13) [ ]

AMD-LIFE: Genetic Risk Score

AMD-LIFE participant number ☐

Your genetic risk score based on common genetic variants: ☐ →

Low	Intermediate	High	Very high
**< -0.057**	**≥ -0.057 en < 1.131**	**≥ 1.131**	**≥ 3**

AMD familial burden:
(1st grade family member or grand parent with AMD)

No	Yes

→ See ' Does a GRS explain all genetic risk in AMD?'

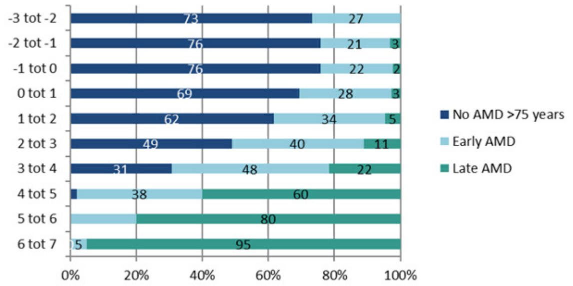

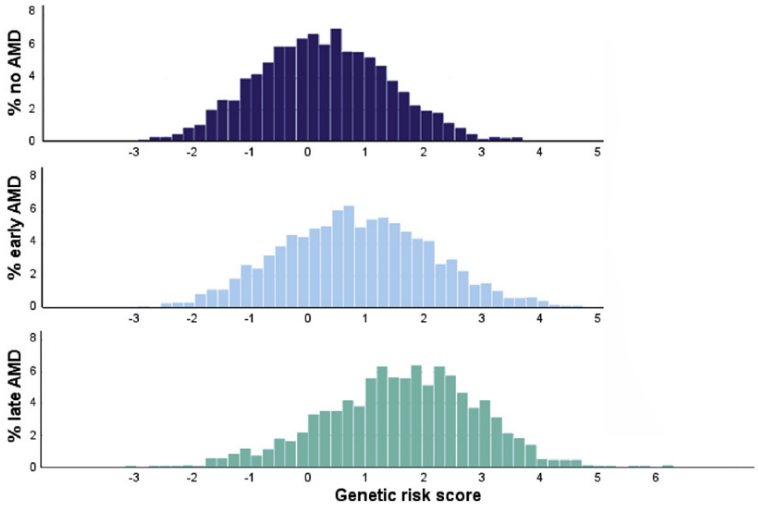

What is a Genetic Risk Score (GRS)?
A genetic risk score indicates how many common genetic variants that increase the risk of age-related macular degeneration (AMD) a person has. The higher the score, the more likely this person will develop late AMD and the greater the risk of blindness. A healthy lifestyle can reduce the genetic risk.

Does a GRS explain all genetics of AMD?
No. Some people have rare genetic variants that greatly increase the risk of macular degeneration. We do not yet have results for these rare variants. You will be notified when we receive it.

AMD-LIFE: The influence of lifestyle explained

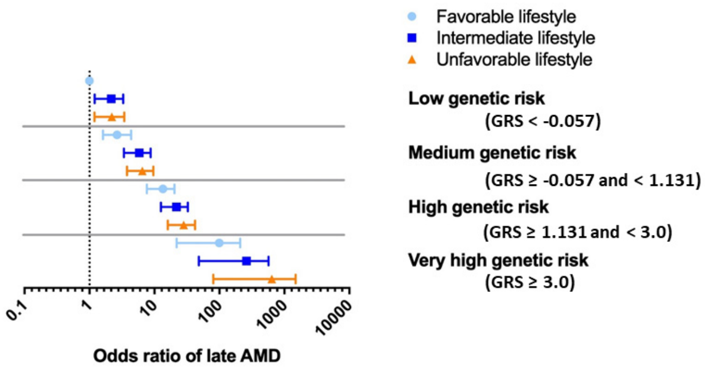

Genetic risk +	Lifestyle ⟶	Higher risk compared to reference
Low	Favorable	Reference
	Intermediate	2 times
	Unfavorable	2 times
Medium	Favorable	2,5 times
	Intermediate	5 times
	Unfavorable	6 times
High	Favorable	12 times
	Intermediate	20 times
	Unfavorable	26 times
Very high	Favorable	67 times
	Intermediate	166 times
	Unfavorable	350 times

The graph and table show the relationship between genetic risk and lifestyle for developing late AMD. A healthy lifestyle (light blue) results in a much lower risk, especially in people with a moderate or strongly increased genetic risk, but there is also a protective effect in people with a low genetic risk.

Reference: Colijn et al, Genetic Risk, Lifestyle, and Age-Related Macular Degeneration in Europe: The EYE-RISK Consortium. Ophthalmology 2021 Jul;128(7):1039-1049

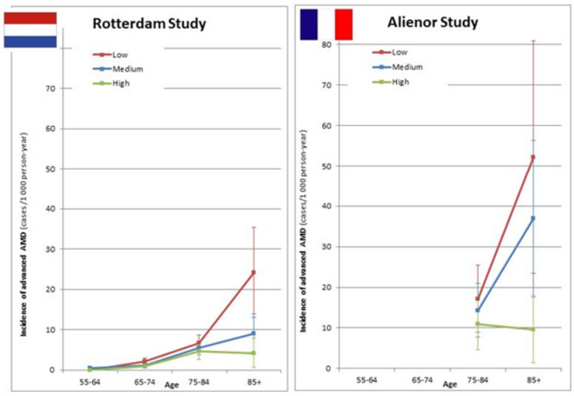

The figure shows the protective effect of a healthy diet on the development of late AMD in two different European studies. Prevalence of AMD is much lower in people with a high score on the Mediterranean diet (green lines) compared to people with low score (red lines). In a Mediterranean diet you mainly use olive oil as fat, you eat a lot of vegetables, fruit, and fish and minimize red meat consumption. This is in line with the Dutch guidelines for healthy diet.

References: Merle et al, Genetic Risk, Mediterranean Diet and Incidence of Advanced Age-Related Macular Degeneration: The EYE-RISK Consortium. Ophtalmology 2019 Mar 126(3) 281-390. De Koning-Backus et al, Intake of Vegetables, Fruit, and Fish is Beneficial for Age-Related Macular Degeneration. Am J Ophthalmol. 2019 Feb;198:70-79

Figure S1. Three page, easy-to-use scoring form used in the AMD-Life study to inform participants on their lifestyle and genetic risk and to stimulate lifestyle change. Page 1: risk estimates for lifestyle factors (i.e., smoking, BMI, diet, physical activity) from validated studies were used to create scoring points. Dietary assessment was performed by a modified version of Mediterranean diet score (wine was excluded; total score 0-13). On the scoring form halves were included to stimulate patients to adherence. Total lifestyle score ranged from 0 to 13 in which high scores represent an AMD-healthy lifestyle. Page 2: Total genetic risk was determined by genotyping the 52 known common risk variants [7] and calculating Genetic Risk Score (GRS) by multiplying the conditional b value of the AMD risk variants with the allele dosage. Subsequently, all calculations were summed. Tertiles of GRS were based on GRS scores of over 17,000 individuals of the EYE-RISK cohort [8] dividing the scores in Low-Medium-High genetic risk. An additional cut-off was added for Very High genetic risk (GRS ≥ 3). Familial burden for AMD (i.e., 1st grade family member or grant parent with AMD) is determined based on questionnaires (No or Yes) to indicate whether rare variants in AMD genes might play a role in the family. Page 3: The interplay between lifestyle and genetic risk is explained by visuals and references to literature.

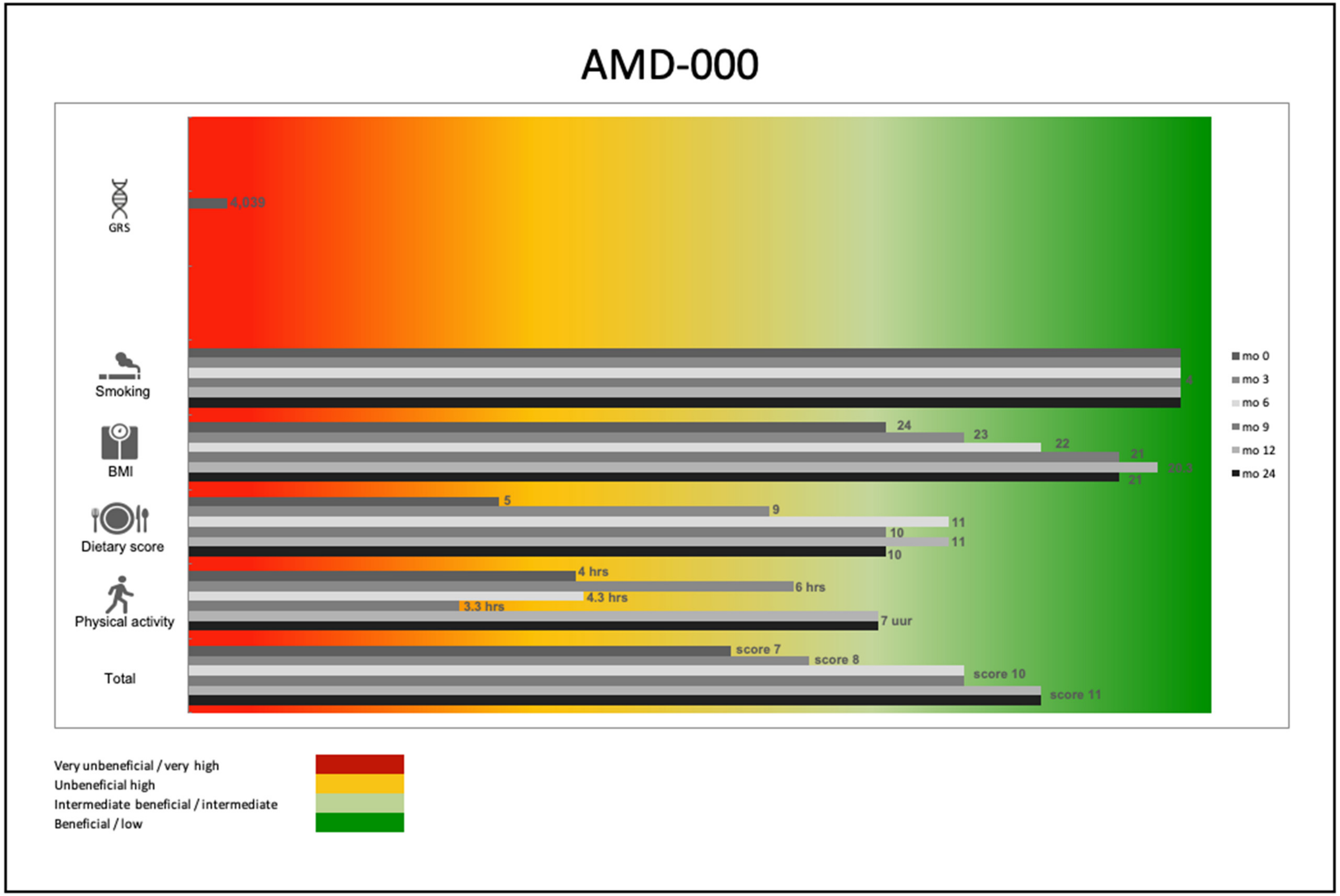

Figure S2. Visualization of the lifestyle scoring over time. The overview graph visualizes the current status of the lifestyle and enables to display follow up measurement.
